# “May-Thurner syndrome as an underlying cause of unilateral left-sided deep vein thrombosis in a young healthy female: A case report”

**DOI:** 10.1016/j.radcr.2024.12.038

**Published:** 2025-01-08

**Authors:** Khaled Sawafta, Fadi Yousef, Hani Abu Hijleh, Wasef Abdat, Yousef Samara, Tasbeeh Al-Kharraz

**Affiliations:** a Department of Medicine, An Najah National University, Nablus, Palestine; b Department of Radiology, Rafidia Surgical Hospital, Nablus, Palestine

**Keywords:** May-Thurner syndrome, Deep vein thrombosis, Iliac vein compression syndrome, Computed tomography angiography, Anticoagulation therapy, Miscarriages

## Abstract

May-Thurner syndrome (MTS), iliac vein compression syndrome, also called Cockett syndrome, is a vascular disease caused by the compression of the left common iliac vein (LCIV) by the right common iliac artery (RCIA) against the lumbar vertebrae. This anatomical defect can lead to venous stasis especially in the left lower limb, and this increases the risk of deep venous thrombosis (DVT). Because routine screening is not standard practice, MTS frequently remains asymptomatic, and its prevalence is probably underestimated. Our case report presents 33 year old women with no thrombotic condition history who complained from a left leg swelling, pain, and stiffness over 5 days. Computed tomography angiography (CTA) confirmed a diagnosis of MTS, and Doppler ultrasonography confirmed extensive DVT in the left lower limb. After receiving conservative treatment with enoxaparin, the patient switched to apixaban therapy. The significance of identifying MTS as a possible cause of unilateral left-sided DVT is highlighted by this case, especially in young, otherwise healthy women. Recurrent DVT and chronic venous insufficiency are among the complications that can be prevented by early detection with imaging and anticoagulation treatment. Patients with atypical DVT presentations may benefit from earlier diagnosis and treatment made possible by greater knowledge of MTS.

## Introduction

May-Thurner syndrome (MTS), also known as iliac vein compression syndrome or Cockett syndrome, is a condition characterized by the compression of the left common iliac vein (LCIV) between the right common iliac artery (RCIA) and the fifth lumbar vertebra. [1]. Prolonged arterial pulsation against the vein leads to intimal fibrosis and the formation of venous spurs, further exacerbating venous outflow obstruction. This compression creates a mechanical obstruction to venous flow, predisposing affected individuals to deep vein thrombosis (DVT) in the left lower extremity (LLE), chronic venous stasis, and venous hypertension. [2].

MTS is often underdiagnosed due to its frequent asymptomatic presentation, with many cases identified incidentally during imaging for other conditions. Despite its asymptomatic nature in many individuals, MTS is a notable contributor to unexplained left-leg DVT, particularly in women aged 30–50 years [3].

While ultrasound has limited utility in diagnosing MTS due to the deep anatomical location of the iliac veins, imaging techniques such as contrast venography or CT venography (CTV) are considered gold-standard diagnostic tools [[4], [10]]. Management strategies depend on symptom severity; asymptomatic cases generally require no treatment, while symptomatic cases with acute DVT are managed with anticoagulation, catheter-directed thrombolysis, and endovascular stenting [5].

This report presents a case of a 33-year-old woman with extensive left-leg DVT caused by MTS, confirmed through CT imaging, highlighting the importance of considering MTS in patients with unexplained lower-extremity DVT.

## Case presentation

A 33-year-old female was in her usual state of health with unlimited exercise tolerance until 5 days prior to admission when she started complaining of left lower leg swelling, pain, and stiffness. The swelling was gradually developing and associated with pain at rest. The patient denied complaining of fever, chest pain, shortness of breath, hemoptysis, or syncope.

Her past medical history includes 3 abortions, a recurrent history of microcytic anemia, and 2 dilation and curettage procedures for abortion. She has no history of deep vein thrombosis (DVT) and no significant family or psychosocial history without any chronic lower limb symptoms.

On physical examination, her heart rate was 85 bpm, blood pressure was 125/74 mmHg, and oxygen saturation was 100% on room air. The patient was conscious, alert, and oriented to time, place, and person, without signs of respiratory distress. Cardiopulmonary examination was unremarkable. The left lower leg examination showed tenderness, swelling, hotness, and erythema, along with palpable bilateral dorsalis pedis pulses and intact sensation without any ischemic changes.

Laboratory tests revealed normal kidney function, serum electrolytes, C3, C4, prothrombin time (PT), partial thromboplastin time (PTT), microcytic hypochromic anemia (HGB 9.5, MCV 63), normal c-reactive protein (CRP) (4), and elevated erythrocyte sedimentation rate (ESR) (40). The ECG and chest X-ray were normal.

**Fig. 1:**
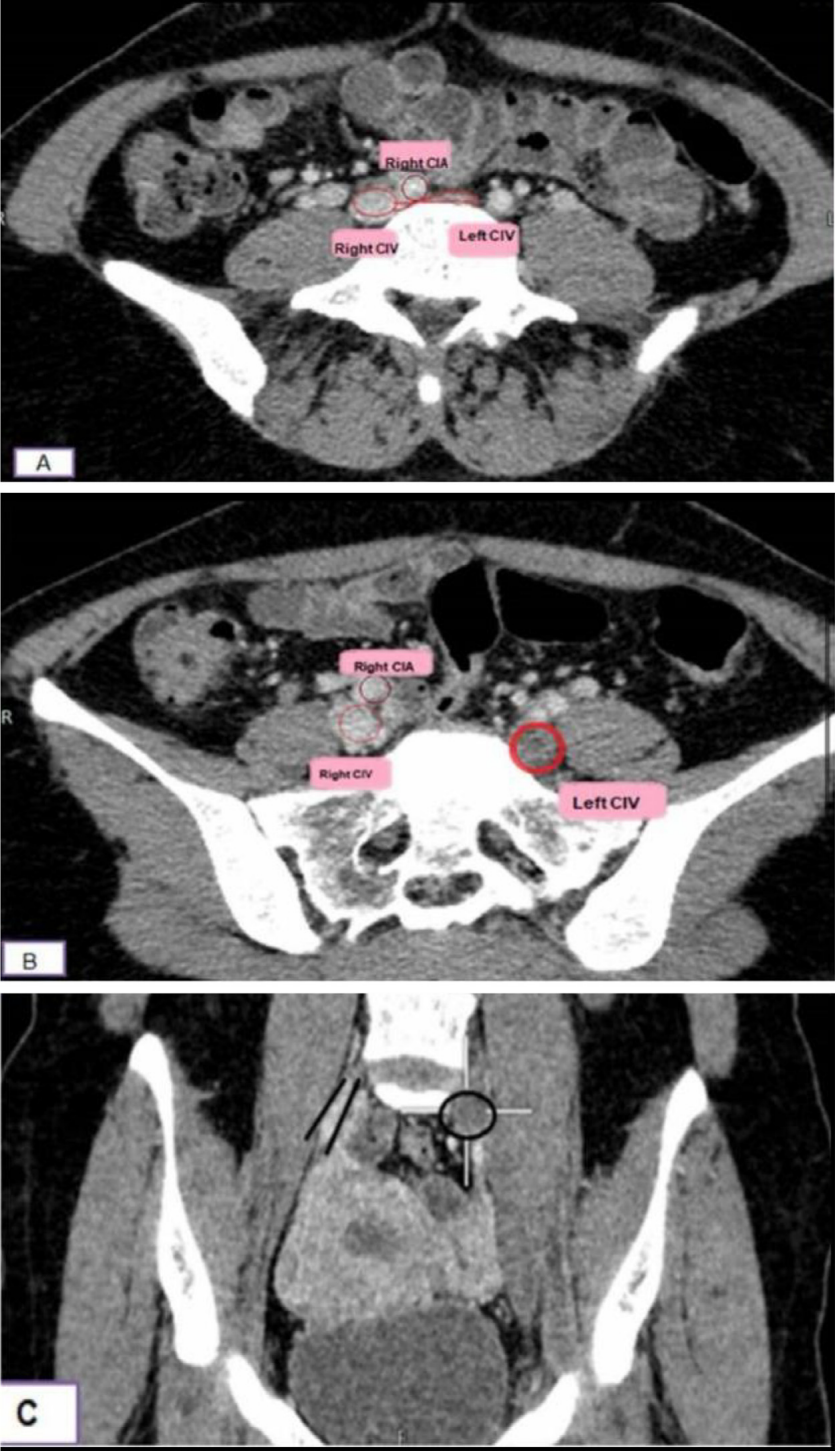
CT Porto-venous phase axial (A, B) and coronal (C). Views: There is compression of the left common iliac vein by the overlying right common iliac artery consistent with May-Thurner syndrome (image A). Filling defect is noted within the dilated, nonenhancing left common iliac vein inferring thrombosis compared to right common iliac vein (image B and C).

On day 5 of symptoms, Doppler ultrasonography of the left lower limb revealed extensive DVT. The porto-venous phase of a computed tomography (CT) scan revealed compression of the left common iliac vein by the overlying right common iliac artery (shown in Fig. 1 below).

Given her history of recurrent abortions along with her presentation with DVT, she was suspected to have antiphospholipid syndrome. Tests for Anti-beta-2-glycoproteins antibodies, lupus anticoagulant, and antiphospholipid antibodies were sent for analysis, which returned negative. In addition to intravenous acetaminophen for pain management, the patient was started on intravenous fluids, omeprazole 40 mg once daily, and enoxaparin 40 mg subcutaneously twice daily.

The patient's history of recurrent abortions and microcytic anemia were initially considered due to their potential links with hypercoagulability. However, the negative antiphospholipid antibody tests made antiphospholipid syndrome less likely as a cause. Microcytic anemia, though recurrent, was attributed to iron deficiency and was not considered a primary factor in the acute DVT. Ultimately, the finding of left iliac vein compression on imaging confirmed May-Thurner syndrome (MTS) as the more significant etiology.

The vascular team was consulted, and the patient was discharged home on omeprazole 40 mg once daily, enoxaparin 80 mg twice daily for 3 days, followed by apixaban 5 mg twice daily.

## Discussion

The patient presented with classic MTS symptoms like many documented cases – unilateral left lower limb pain, swelling, and tenderness—consistent with the known prevalence of MTS among women's ages between the 30s and 50s, with most cases being asymptomatic or manifest with generalized and nonspecific symptoms that will go unnoticed until more serious complications like DVT or PE develop [[6], [7]]. Therefore, it is crucially important to be aware that MTS might go unnoticed and subsequently lead to the development of more serious complications. This highlights the importance of increasing awareness of MTS, particularly in at-risk populations.

Diagnostically, the case was diagnosed using standard tools. This includes Doppler ultrasound, usually unsatisfactory given their deep anatomical location—the iliac veins. Therefore, invasive investigation through CT angiography was accomplished for the purpose of good diagnosis and showing the compression of the left common iliac vein by the overlying right common iliac artery. Confirming the definite need for further advance on noninvasive imaging to appropriately handle such kinds of presentation. The conservative treatment approach taken, using oral anticoagulation like enoxaparin and apixaban alone, was effective in the prevention of recurrences and avoidance of more aggressive therapies, such as endovascular stenting, which may be considered in cases of significant venous compression, venous HTN history of recurrent DVT or when conservative approach fails [[7], [8], [9]].

The development of swelling, pain, and tenderness in the left lower extremity, added to the fact that there was no other possible cause for her DVT, further provides interest in pointing out MTS as one of the differential diagnoses when unilateral left lower limb DVT occurs without an obvious precipitating factor. She may have been more susceptible to DVT in the presence of MTS due to her history of microcytic anemia and multiple miscarriages, which could indicate a hypercoagulable state. However, MTS is most likely the main cause of her recent DVT, given her negative vasculitis profile, lack of previous DVT, and lack of acquired or hereditary thrombophilia. This case emphasizes how MTS should not be ruled out even if a history of thrombotic events is not known. Two imaging techniques were used to make the diagnosis: computed tomography angiography (CTA) and Doppler ultrasonography. Particularly when applied in this case, CTA demonstrated in great detail the anatomic relationship of the iliac vessels and was crucial in confirming the diagnosis in addition to that it is not invasive, in some cases we may need to use additional imaging techniques such as venography but as it's invasive we thought of it as a last resort when the diagnosis is uncertain or when we want to do a simultaneous intervention like stenting.

## Clinical significance

May-Thurner syndrome (MTS) is a relatively unknown and sometimes ignored diagnosis. However, it is a major cause of left lower extremity deep vein thrombosis (DVT). This instance emphasizes the necessity of increasing physician and healthcare provider knowledge of MTS. As noted in various pieces of literature, MTS can lead to serious complications if left untreated, This includes chronic venous insufficiency and post-thrombotic syndrome, one of the most common complications of MTS. The clinical significance mainly lies in the importance of considering MTS in the differential as a cause of recurrent or unexplained left leg DVT, as early identification is extremely important and can greatly influence the management strategies. Early identification of MTS changes our approach from standard anticoagulation to more specific interventions. Moreover, early intervention will help reduce complications, including post-thrombotic syndrome, to a rate of less than 10% [10]. Moreover, asymptomatic or general symptoms of MTS are a big challenge in clinical practice and require detailed evaluations for the early detection of this anatomical variant [9].

## Limitations

Despite the insights acquired from this case study, certain limitations must be addressed, which may impact the findings' interpretation and application. One of the main limitations of this study is the fact that it is a single case report. These findings therefore cannot be generalized to a greater population of patients with MTS, since there is great individual variation in the presentation of symptoms and response to treatment. Moreover, this case report was based on a retrospective form of study of the patient's course of illness and treatment, which might introduce some unintentional bias in the interpretation of symptoms. Yet another limitation faced within this case was due to the lack of diagnostic tests required for MTS, as it relies on advanced imaging techniques, which resulted in the use of an invasive form of imaging that could delay diagnosis. Furthermore, the outcome results that were provided in this study were short-term outcomes, and further follow-up would help provide a better understanding of the effectiveness of the treatment plan implemented. Other limitations in this study might include the potential underreporting of the condition's prevalence and complications, mainly due to the nature of the disease being asymptomatic. Besides, without the control group, there is also no clear comparison of the success of the treatment plan executed here.

## Future directions

The findings of this case report highlight the need for more study into numerous critical areas connected to May-Thurner Syndrome (MTS). This includes the need for more studies that focus on the long-term outcomes of patients diagnosed with MTS in order to evaluate the efficacy of each treatment strategy. It also brings to light the establishment of a uniform MTS screening protocol in patients with unprovoked or recurrent left leg DVT. This will be of value, especially in populations where the risk is high. Another possible direction for future research is the exploration of the effectiveness of varying imaging modalities, as this can help improve early detection of MTS [1]. More studies are still needed to have a better understanding of the natural history of asymptomatic cases and how missed diagnoses can be

## Conclusion

This case emphasizes the importance of identifying May-Thurner syndrome (MTS) as a potential underlying cause of unexplained left-sided DVT, particularly in women of reproductive age. MTS should be considered in patients with unilateral left lower limb DVT without apparent risk factors. Effective anticoagulation management is critical to reducing recurrence and preventing chronic venous insufficiency. Timely evaluation and increased awareness are essential to lower morbidity associated with undiagnosed venous compression syndromes.

## Patient consent

Written informed consent was obtained from the patients for their anonymized information to be published in this article.

## Ethics approval

Our institution does not require ethical approval for reporting individual cases or case series.
